# Liver-Targeting Nanoplatforms for the Induction of Immune Tolerance

**DOI:** 10.3390/nano14010067

**Published:** 2023-12-26

**Authors:** Sydney Kusumoputro, Christian Au, Katie H. Lam, Nathaniel Park, Austin Hyun, Emily Kusumoputro, Xiang Wang, Tian Xia

**Affiliations:** 1 Department of Medicine, Drexel University College of Medicine, Philadelphia, PA 19129, USA; skk78@drexel.edu (S.K.); njp86@drexel.edu (N.P.); 2 Department of Ecology and Evolutionary Biology, University of California, Los Angeles, CA 90095, USA; 3 Department of Bioengineering, University of California, Los Angeles, CA 90095, USA; cjau@usc.edu; 4 Department of Medicine, Keck School of Medicine, University of Southern California, Los Angeles, CA 90007, USA; khlam@usc.edu; 5 Department of Microbiology, Immunology, and Molecular Genetics, University of California, Los Angeles, CA 90095, USA; 6 Department of Medicine, Lake Erie College of Osteopathic Medicine, Bradenton, FL 34211, USA; ahyun98275@med.lecom.edu; 7 Department of Integrative Biology and Physiology, University of California, Los Angeles, CA 90095, USA; 8 Department of Evolution, Ecology, and Organismal Biology, University of California, Riverside, CA 92521, USA; ekusu001@ucr.edu; 9 Division of NanoMedicine, Department of Medicine, University of California, Los Angeles, CA 90095, USA; 10 California NanoSystems Institute, University of California, Los Angeles, CA 90095, USA

**Keywords:** immune tolerance, liver-targeting nanoparticles, autoimmune diseases, allergies

## Abstract

Liver-targeting nanoparticles have emerged as a promising platform for the induction of immune tolerance by taking advantage of the liver’s unique tolerogenic properties and nanoparticles’ physicochemical flexibility. Such an approach provides a versatile solution to the treatment of a diversity of immunologic diseases. In this review, we begin by assessing the design parameters integral to cell-specific targeting and the tolerogenic induction of nanoplatforms engineered to target the four critical immunogenic hepatic cells, including liver sinusoidal epithelial cells (LSECs), Kupffer cells (KCs), hepatic stellate cells (HSCs), and hepatocytes. We also include an overview of multiple therapeutic strategies in which nanoparticles are being studied to treat many allergies and autoimmune disorders. Finally, we explore the challenges of using nanoparticles in this field while highlighting future avenues to expand the therapeutic utility of liver-targeting nanoparticles in autoimmune processes.

## 1. Introduction

Autoimmune diseases and food allergies are caused by adaptive immune responses to self- and food antigens, respectively. The prevalence of these conditions in the United States is significant, with approximately 24 million people suffering from autoimmune diseases and 15 million suffering from food allergies in 2021 out of a total population of 331.9 million people. Currently, available therapeutic options, such as immunomodulatory drugs, monoclonal antibodies, and anti-inflammatory drugs, carry the potential for increased susceptibility to opportunistic infections or interference with cancer screenings due to their systemic effects [1,2]. As such, there is a pressing need for the development of more effective immune therapies that can promote sustained immune tolerance and have high specificity for target molecules.

The liver, which functions as an immune-privileged organ with tolerogenic properties, is a popular target for such a sustained immune response. Due to its function and anatomical location, the liver is constantly exposed to a wide variety of dietary, commensal, and exogenous antigens. As such, it naturally mediates peripheral tolerance towards self- and foreign antigens and contains cells that function as anti-inflammatory mediators [3,4,5]. Thus, the liver plays an integral role in preventing an immune response towards innocuous antigens, including commensal bacterial metabolites, food-derived antigens, and other harmless pathogens [6,7].

**Figure 1:**
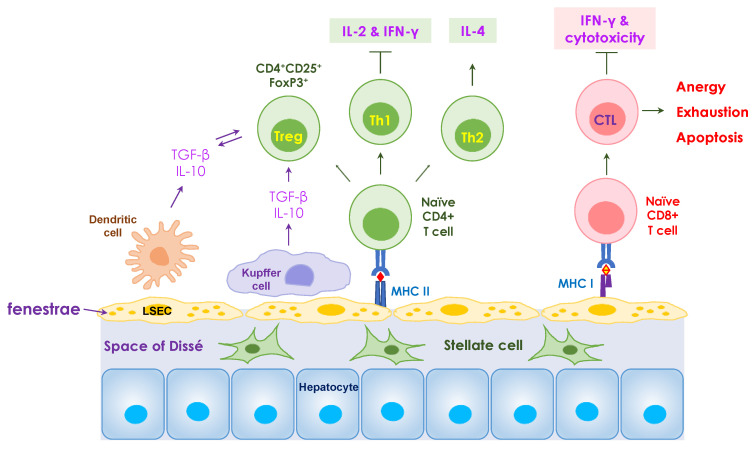
**Mechanisms of tolerance mediated in the liver**. Liver sinusoidal endothelial cells (LSECs) play a critical role in hepatic immune surveillance by clearing antigens in the blood. The fenestrae of LSECs allow the penetration of nutrient or antigen molecules into the subendothelial space of Dissé. Antigen presentation by LSECs to naïve CD8^+^ T cells results in the development of dysfunctional cytotoxic T lymphocytes (CTLs) to induce cytotoxicity, including anergy, exhaustion, and apoptosis. The interaction between LSECs and naïve CD4^+^ T cells induces the differentiation of naïve T cells towards Th2 and regulatory T (Treg) cell phenotypes and suppresses cytokine production by Th1 cells. Kupffer cells, in conjunction with LSECs, secret IL-10 and TGF-β, provide a tolerogenic microenvironment locally in the liver. The resident liver dendritic cells also produce IL-10 and TGF-β and play an important role in tolerance induction.

**Figure 2:**
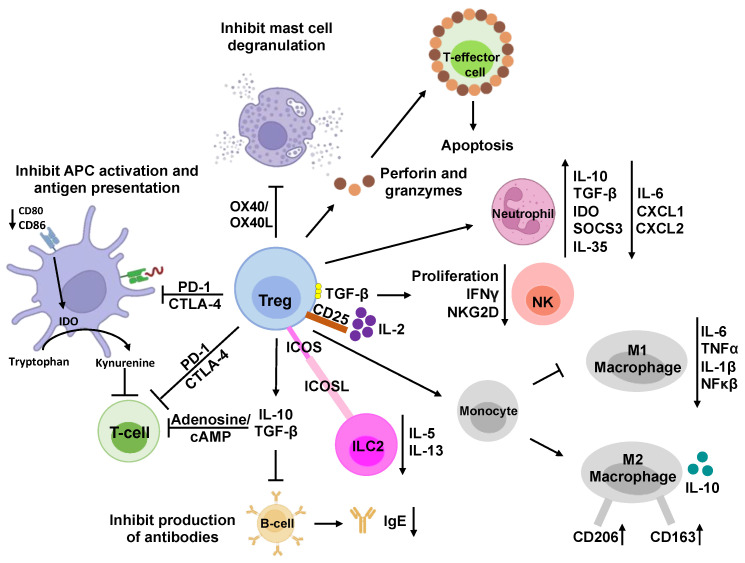
**Immunosuppressive mechanisms of regulatory T cells**. Regulatory T cells (Tregs) are a subset of CD4^+^ T cells that modulate the immune system and prevent allergies and autoimmune diseases. They suppress the activity of various immune cells and maintain immune homeostasis through direct and indirect mechanisms. Indirectly, Tregs can secrete anti-inflammatory cytokines, such as IL-10, IL-35, and TGF-β, to inhibit the activation and proliferation of T-effector cells and NK cells. They can also release perforin and granzyme to induce cell death, i.e., apoptosis, by creating pores in the target cell membrane and activating caspases. In addition, Tregs can suppress immune cells by interacting with them directly. For example, Treg cells express high levels of CD25, which is the IL-2 receptor, to sequester IL-2 from the microenvironment, reducing the growth and function of T-effector cells and NK cells. Tregs can also affect NK cells directly by presenting membrane-bound TGF-β to them. Moreover, Treg cells can modulate B cells and dendritic cells by engaging with PDL1/PD-1 and CTLA-4/LAG-3 pathways, respectively. CTLA-4 can also block co-stimulation by reducing CD80/CD86 expression on dendritic cells and induce the upregulation of IDO, which degrades tryptophan and inhibits T-cell proliferation. Additionally, Tregs can express CD39, which converts ATP to adenosine and AMP and reduces T-effector-cell proliferation. Furthermore, Treg cells can skew monocyte differentiation toward anti-inflammatory M2 macrophages and prevent them from becoming pro-inflammatory M1 macrophages. Similarly, Treg cells can induce a suppressive phenotype in neutrophils and reduce ILC2 cytokine secretion. Tregs can make direct contact with mast cells and basophils through OX40/OX40L interaction, modulate their phenotype and activity, and inhibit the release of histamine and IgE production.

The liver cells specifically involved in perpetuating the tolerogenic environment include liver sinusoidal epithelial cells (LSECs), Kupffer cells (KCs), hepatic stellate cells (HSCs), dendritic cells, and hepatocytes [8]. Some of these cells function as antigen-presenting cells (APCs) and dampen T-cell responses by increasing the number of regulatory T cells (Tregs); inducing negative co-stimulatory molecules, CTLA-4 (cytotoxic T-lymphocyte-associated protein 4) and programmed-death ligand 1 (PD-L1); producing immune-modulatory cytokines, interleukin (IL)-10, IL-35, and transforming growth factor-beta (TGF-β); and inhibiting effector cells’ function of adenosine generation. For example, as APCs, the LSECs contain major histocompatibility complexes (MHCs) that recruit T lymphocytes and present antigens to CD4^+^ and CD8^+^ T-helper cells for appropriate immune responses (Figure 1). Specifically, CD4^+^CD25^+^FoxP3^+^ regulatory T cells (Tregs), largely considered the dominant natural Treg population, play a critical role in maintaining immune tolerance and homeostasis of the immune system by suppressing cell–cell interactions and producing immunosuppressive cytokines, including IL-10, IL-35, TGF-β, etc. Consequently, the complex interactions among liver cells and the production of anti-inflammatory cytokines contribute to the induction of adaptive immunogenic tolerance in the hepatic microenvironment (Figure 2) [8,9]. A platform that can, thus, take advantage of these beneficial tolerogenic mechanisms would have a huge impact on the development of sustainable therapies for allergies and autoimmune diseases.

The current landscape of allergy and autoimmune disease therapies, however, is marked by significant limitations. For one, conventional treatments often focus on alleviating symptoms rather than addressing the underlying immune dysregulation and are, thus, only temporizing measures [10]. Supportive treatment for anaphylaxis includes epinephrine, antihistamines, and corticosteroids. Similarly, corticosteroid treatment is employed, for instance, in managing conditions like autoimmune hepatitis. Additionally, long-term immunosuppressive therapies, such as TNF-**α** inhibitors for multiple sclerosis, are often key players in autoimmune therapies but also provoke compromised immune systems susceptible to common and opportunistic infections [11]. To address this concern, many studies have demonstrated antigen-specific immune tolerance, which can alleviate the overactive immune responses in allergic disorders and autoimmune diseases without global immunosuppression [12]. However, because they also lack targeting specificity within the body, they are still associated with widespread adverse effects, excessive therapeutic doses, and a higher risk of infection and cancer [2]. The challenge, therefore, lies in achieving a targeted and specific modulation of the immune system to induce long-lasting tolerance without systemic effects. However, the unpredictable nature of allergen exposure and variations in individual immune responses make it challenging to provide preemptive measures effectively [10].

Nanoparticles (NPs) have emerged as a promising approach for the induction of immune tolerance with their unique physicochemical properties and specific targeting abilities [13,14]. Innovative approaches in nanoparticle designs have led to the construction of new formulations of nanoplatforms that can mediate tolerogenic responses and target specific immunomodulatory cells [15]. By taking advantage of the liver’s tolerogenic mechanisms, the antigen-specificity that NPs can offer, and the NPs’ functional versatility, liver-targeting NPs can provide focused and effective immunosuppression. In this literature review, we assess the design parameters integral to cell-specific targeting and immune tolerance induction and discuss how such nanoplatforms can be engineered to target the four relevant hepatic cells to induce immunogenic peripheral tolerance. Finally, we examine multiple therapeutic strategies in which NPs can be used to treat hypersensitivity reactions, including allergic reactions and autoimmunity.

## 2. Nanoparticle Features That Impact Liver-Targeting and Tolerogenic Effects

**Figure 3:**
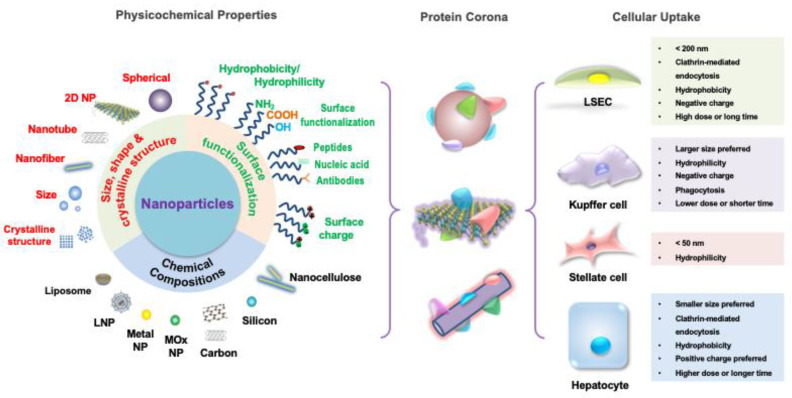
**NP features and designs for hepatic cellular targeting**. The different features of nanoparticles individually contribute to the particles’ unique biochemical activity but also act synergistically to target the different tolerogenic hepatic cells. The composition of the nanoparticle can be of inorganic materials, such as metals and carbons, or organic materials, such as lipids and polymers, which influences their tolerance induction capability. Nanoparticle size, shape, and crystallography regulate biodistribution, cellular internalization, and cellular clearance. Surface functionalization influencing hydrophilicity, valence, ligand-specific binding ability, and biomimicry impact cell-particle interactions with targeting, activating, and inhibiting effects. Thus, by selecting the appropriate combination of features, a diversity of nanoparticles that target the hepatic cells according to each one’s unique cellular uptake preferences can be designed.

The synthetic versatility of nanoparticles and the opportunity to fine-tune their physicochemical properties have implored their use as novel platforms for antigen-specific immune tolerance induction. In this regard, some nanoparticles have been implemented to manipulate antigen presentation pathways while others directly interfere with the function or elimination of antigen-specific immunological signaling [2,16]. Nanoparticles’ ability to penetrate cells, such as phagocytic cells, also makes them an attractive solution to the hydrophilicity barrier [17]. In these cases, there is a range of physical parameters (e.g., size, shape, surface charge, and composition, etc.) and functional modifications (e.g., surface modifications and payload encapsulation, etc.) that can be bioengineered to provide different advantages, as illustrated in Figure 3. Additionally, since most intravenously administered nanoparticles show natural liver accumulation, they are intrinsic liver-targeting platforms for immunomodulation [18]. Engineering to achieve passive targeting, active targeting, and therapeutic delivery can, therefore, be integrated into nanoparticle designs for the development of liver-targeting tolerance induction nanoplatforms.

### 2.1. Size and Shape

The evolution of the immune system to sense and capture particulates, such as microbes and microbial products, on the nanometer scale initially entreats the use of synthetic nanoparticles for tolerance induction [19,20]. Therefore, when considering immune system targeting, and in particular liver targeting, particle size and shape are fundamental design parameters. They are especially important when considering “passive targeting” of the liver, or the manipulation of physical parameters to influence nanoparticle pharmacodynamics and targeting. Size and shape will not only affect kinetics regarding bloodstream circulation, cellular clearance, and intracellular transport but also contribute to nanoparticles’ biodistribution and cellular internalization [21].

In terms of biokinetics, it has been observed that the smaller the hydrodynamic diameter of the nanoparticle, the more widely distributed throughout the body the nanoparticle becomes [22]. This has been attributed to smaller particles being less prone to marginalize in normal laminar blood flow, having a higher propensity to accumulate within all types of cells, and having decreased cellular clearance [20,21]. Multiple studies also support this observation, with the convergence of intravenously delivered nanoparticle size towards an ideal diameter range of 30–300 nm for maximum particle circulation [21]. Likewise, it has been observed that as particle size increases, there is an increased accumulation in the liver and spleen, both of which have sinusoidal basal lamina allowing for permeability and the former of which is the largest cardiac-output-receiving organ at rest [23].

Specific liver-residing cells also display particle size preferences due to the impact of size on cellular internalization rates and mechanisms. For example, LSECs have been shown to engulf particles with a diameter of up to 200 nm due to their internalization method of receptor-mediated endocytosis [24,25]. This upper size limit is shown to facilitate intracellular accumulation in late endosomal and lysosomal compartments, an important mechanism for immunomodulation, within other non-phagocytic cells [25]. On the other hand, Kupffer cells, which can also utilize receptor-mediated endocytosis, preferentially uptake particles > 200 nm in the liver due to their phagocytic activity [25,26]. This creates a well-controlled dichotomy that makes up most of the liver’s scavenger function. To access other liver cells, such as hepatocytes and HSCs, penetration through the liver sinusoidal fenestrae via the space of Dissé may require minimization of nanoparticle size, ideally to <100 nm [27]. However, it has been shown that nanoparticles up to 400 nm have been able to extravasate into the space of Dissé with a proper lipid composition of negatively charged phosphatidylserine [28].

Regarding shape, spherical nanoparticles are the most common particle shape and are shown to have some preferential accumulation in the lungs, liver, and spleen [21]. Most studies have additionally shown increased phagocytosis of spherical nanoparticles by macrophages compared to elongated ones [29]. PEG-*bl*-PPS micelle nanoparticles, for example, were shown to preferentially target liver phagocytes compared to more elongated fillomicelle nanoparticles [30]. Contrastingly, however, cylindrical silicon-based nanoparticles showed nearly two times higher accumulation in the liver than their spherical counterparts [31]. Therefore, while there is a strong impact of size and shape on liver targeting and biodistribution, more research needs to be conducted to discern their specific targeting impact and the varying influence different nanoparticle compositions may have on such impact. Furthermore, nanoparticle size may dictate the contribution of shape towards cellular accumulation, as seen with rod-shaped nanoparticles, which have shown higher uptake efficiencies within a certain range of aspect ratios and lengths (e.g., 200–300 nm for nanocellulose NPs) [32,33].

When discussing size and shape, it is also important to note their contribution to toxicity. A review of the literature suggests that particles smaller than 10 nm may exhibit toxic effects due to inefficient cellular clearance and prolonged accumulation while those larger than 1000 nm may exhibit toxicity secondary to capillary obstruction [21]. Similarly, certain shapes, such as needle-like particles, have been shown to damage lysosomal membranes, inducing NLRP3 inflammasome activation and instigating inflammatory responses due to characteristics such as their rigidity and propensity to shed toxic ions [33,34]. These overarching safety parameters must be an important consideration during nanoparticle design.

### 2.2. Surface Charge

Another important physical parameter that contributes to the nanoparticle passive targeting of the liver, especially at the cellular level, is surface charge. While cationic nanoparticles have shown greater safety and biocompatibility concerns due to their strong membrane permeability and binding of negatively charged DNA, deviations towards either a positive or negative surface charge have shown significant effects on particle–cell interactions [35,36]. Highly cationic or anionic nanoparticles, for example, adsorb a significant amount of serum protein, resulting in aggregation and increased uptake by macrophages in vitro [37]. Kupffer cells and LSECs have also been shown to preferentially bind and clear negatively charged nanoparticles through interactions with scavenger receptors, such as stabilin-1 and stabilin-2 [38,39]. Conversely, hepatocytes demonstrate significant uptake of positively charged NPs over negatively charged ones [39]. The desired zeta potential for enhanced and targeted membrane interactions can be achieved either through material selection of choice, as in the case of many ionizable lipids, or chemical modifications [40]. However, it is important to note that protein binding to, e.g., albumin, formation of protein coronas, and interactions with phagocytes can change the synthetically given surface charge in vivo and subsequently influence liver cellular uptake [29,41]. Therefore, despite the role that electrostatic binding plays in liver uptake, the interplay is complex between the nanoparticles’ different characteristics that ultimately determine their activity.

### 2.3. Composition

Another advantage of nanoparticles is the diversity of materials from which they can be manufactured to optimize their desired function and payload compatibility. Four broad categories of materials used for nanoparticle synthesis include metals, lipids, carbon-based materials, as well as synthetic and natural polymers [2].

Metal and metal-oxide nanoparticles have long been implemented for theranostics and have recently been designed to conjugate target ligands, antigens, and immunomodulators on their surfaces. A few examples for the treatment of autoimmune diseases, such as experimental autoimmune encephalomyelitis (EAE) and type I diabetes (T1D), include iron oxide NPs, which have been popularly conjugated to high-avidity MHC peptide ligands, and gold NPs, modified with T-cell epitopes and 2-(1′H-indole-3′-carbonyl)-thiazole-4-carboxylic acid methyl ester [42,43,44]. Gold and silicon nanoparticles, amongst others, have also been designed for various therapeutic applications [45]. However, the typical requirement for payloads to be conjugated to the nanoparticle metal surface may severely limit the types of molecules and, thus, applications of such particles [2]. Innocuous antigens and/or their epitopes, for example, may be difficult to implement with such metal or metal-oxide nanoparticles for antigen-specific tolerance. Additionally, while the stability of these particles can be highly favorable depending on the application, their lack of biodegradability has been demonstrated to increase tissue accumulation, creating toxicity and safety concerns [2,46].

Lipid-based nanoparticles (LNPs) are another popular nanoparticle of choice and have been clinically used for a variety of FDA-approved applications, ranging from ONPATTRO^®^ polyneuropathy therapy to the mRNA-1274 (Moderna, Cambridge, MA, USA) and BNT162b2 (Pfizer-BioNTech, New York, NY, USA) COVID-19 vaccines [47,48]. One reason for this is their high degree of biocompatibility and their reduced risk of producing inflammatory responses due to lipids’ status as a natural component of the cell membrane [21]. Another advantage is the ability of liposomal NPs to encapsulate hydrophobic molecules in their lipid bilayer, hydrophilic molecules in their aqueous core, or both simultaneously, giving them compatibility with diverse payloads. Conversely, other lipid-containing NPs incorporate hydrophobic molecules into the lipid construct before mixing with the aqueous phase for NP synthesis [49]. LNPs have been shown to deliver not only small molecules, such as anti-cancer drugs and chemotherapy agents [50,51], but also, more recently, nucleic acids. LNPs encapsulated with siRNA targeting ApoB [52], PCSK9 [53], PLK1 [54], VEGF, and KSP [52] expression are just a few examples, along with those delivering self-amplifying RNA (saRNA) [55] and mRNA [50,51,52,53,54,55]. The enhanced flexibility for payloads of diverse chemical properties also allows opportunities for the co-delivery of molecules, such as a combination of antigenic material with tolerogenic agents (e.g., suppressive cytokines and broad immunosuppressants) [56]. Additionally, other LNPs have been surface-modified with engineered peptides and whole membrane proteins, providing further avenues for their possible use as antigen-specific immune tolerance therapeutics [57]. Finally, a low-shear extrusion manufacturing method can minimize the protein denaturation risk during encapsulation. However, payload release kinetics may be difficult to fine-tune with liposomes [1].

Liposomes may also experience a slight propensity to targeting. This has been partially attributed to their cholesterol-dependent membrane fluidity that enables the clustering of surface molecules upon interaction with the target cell [2]. As such, there has recently been research and development into passively and actively targeting LNPs in the liver through engineering their physicochemical properties and taking advantage of surface modifications by targeting ligands [58]. Lipid-coated calcium phosphate NPs, for example, have been extensively studied in their targeting of hepatocytes and HSCs to deliver siRNA [59]. The successful use of LNPs in ONPATTRO, the first FDA-approved liver-targeting nanomedicine, in 2018 validates such widespread usage and further development of LNPs for liver targeting [47].

Carbon-based NPs, such as carbon nanotubes and fullerene, have mostly been implemented to promote or suppress immune responses as adjuvants, showing various results in antigen-based immunotherapy (AIT), with carbon nanotubes worsening systemic allergic reactions and carbon allotropes reducing anaphylaxis [59,60]. Conclusively, however, observations of high levels of toxicity and detrimental health effects have been attributed to their failure to degrade naturally [61]. Therefore, extensive investigation into their persistence, bioaccumulation, and toxicity in the human body would be further required before implementation.

Finally, natural and synthetic polymers have been popularly used for therapeutic NP manufacturing, notably in the field of tolerance induction [60]. Their synthetic diversity over their physicochemical properties and their proclivity for surface modifications, such as receptor ligands, make them apt to target specific liver-residing cells [60,61]. Therapeutically, their encapsulation of antigens provides a physical protective barrier from the human body environment and possible IgE binding-causing anaphylaxis [60]. Their long shelf-life is another important factor for clinical usage [21].

Polymeric nanoparticles can also be split into non-degradable NPs and biodegradable NPs. Non-degradable NPs that have been researched for immune modulation mainly consist of dendrimers, such as allergen-encapsulating poly(ethylene glycol) (PEG), and benefit from their design flexibility, multivalent surfaces for ligand surface modification, and enhanced solubility and bioavailability [60,61].

Biodegradable polymers, on the other hand, provide a safe, biocompatible material for nanoparticles, many of which have already been approved in medical devices and drugs. Furthermore, the choice of polymer and composition can strongly affect the payload release rate [62,63]. Natural biodegradable polymers, such as chitosan, alginate, carrageenan, albumin, gelatin, collagen, and mannan, have been implemented as allergen carriers for relatively quick antigen release while synthetic biodegradable polymers, such as polylactic acid (PLA), poly lactic-co-glycolic acid (PLGA), poly(methyl methacrylate), polycaprolactone, poly(alkyl cyanoacrylates), and copolymers, have enabled extended release over days or weeks [60]. The selection of material, thus, endows a fine-tuned control over the release rate that may be necessary for AIT therapies requiring a short or long treatment period. Amongst these, PLGA is the most utilized and extensively studied NP material for tolerance induction due to its strong biocompatibility, biodegradability, nontoxicity, and FDA approval for drug delivery [64,65]. These have been demonstrated in tolerance towards EAE, rheumatoid arthritis, T1D, etc. [64]. Overall, while the unique advantages and disadvantages of different manufacturing materials must be carefully chosen for the desired tolerance applications, the diverse selection provides a wide array of modifications and opportunities for tolerance induction.

### 2.4. Ligands and Modifications

As previously discussed, modifications and ligand conjugations to nanoparticles, regardless of composition, can be important features in the targeting design (targeting ligands) and therapeutic action (alloantigens, activating and blocking ligands, biomimicry, and therapeutic cargo) of nanoparticles. Targeting ligands, such as mannose, ApoB peptide (RLYRKRGLK with GCC tag), and hyaluronic acid, amongst others, are vital for directing nanoparticles toward desired liver-residing cell populations [66]. This is especially important to increase their potency and safety [59]. Alloantigens, including ovalbumin (OVA), myelin oligodendrocyte glycoprotein (MOG), and proteolipid protein (PLP), will also be discussed as they can be either loaded or surface conjugated as the payloads to be delivered for AIT and autoimmune treatments [67]. Possible conjugations to nanoparticles, however, expand beyond these.

Activating ligands, for example, have also been accessorized onto NPs to target tolerogenic receptors. Gold NPs conjugated with aryl hydrocarbon receptor (AHR) ligands have been shown to induce FoxP3^+^ and IL-10^+^ Treg and Th17 differentiation [44,68]. Similar ligands have also demonstrated effector cell killing effects; polymeric latex bead NPs conjugated to Fas receptor monoclonal antibodies deleted antigen-specific cytotoxic T cells and LNPs displaying CD22 glycan ligands and antigens caused the apoptosis of human B cells [69]. Meanwhile, NPs decorated with blocking ligands have shown anergic and “sponge”-like results. The addition of antigen peptide-bound MHC I or MHC II on NPs without costimulatory signals, for example, has converted CD8^+^ T cells into regulatory, anergic phenotypes and also increased the production of Tregs, IL-10, and regulatory B cells [42,43]. Likewise, the adornment of macrophage membrane antigens TNFR2, CD36, and CCR2 onto chitosan NPs has caused the sequestration of the pro-inflammatory cytokines TNF-alpha and IL-1β [70]. The latter nanoplatform, which mimicked the macrophage membrane’s anti-inflammatory characteristics, is one example of the rapidly dividing research on biomimetic NPs that take advantage of numerous modifications.

A major advantage to utilizing NPs for tolerance in transplantation, autoimmunity, and allergies is that they can simultaneously functionalize a set of antigens or antibodies onto the surface of the same NP to achieve biomimicry. Surface modifications with such proteins derived from biological sources endow nanoparticles with complex cellular functions, such as adhesion, targeting, and signaling, while maintaining loading abilities [19]. To capitalize on this, NPs have been engineered to mimic a diversity of biologics, such as leukocytes, chromatin, dead cells, and platelets [2,42,71,72]. Leukosomes have been designed to conjugate leukocyte ligands, including LFA-1, CD45, CD47, PSLG-1, Mac-1, etc., to increase the expression of IL-10 and TGF-β genes for immunomodulation [21,71]. Alternatively, DNA-protein nanocomplexes mimicking chromatin’s tolerogenic function to induce the tolerogenic phenotype in APCs have induced tolerance towards its OVA and keyhole limpet hemocyanin (KLH) cargos [72]. This tolerogenic APC phenotype can be further taken advantage of by other nanoparticles that use dead-cell peptide conjugates to mimic dying cells with the purpose of inducing apoptosis and antigen-specific tolerance [2]. Liposomes decorated with phosphatidylserine, for example, have been shown to bind macrophage PS-specific scavenger receptors, promoting tolerogenic phenotypes for hemophilia A and Pompe disease treatment [73,74].

Since cargo is another important feature of NP design for therapeutic effects, it is also important to note that NPs have co-administered protein antigens and immunosuppressive drugs, such as NF-kB and mTOR inhibitors [2]. Immunomodulatory cytokines have likewise been loaded into and conjugated onto nanoparticles. TGF-β, for example, was conjugated onto antigen-loaded PLGA NPs to improve their tolerance induction efficacy [75].

Overall, the ability to fine-tune NPs’ physicochemical properties of size, shape, and surface charge; pick from a wide selection of materials; and diversely modify ligands and payload positions NPs as a strong platform for liver-targeting tolerance induction.

## 3. Liver Cell Targets

### 3.1. Liver Sinusoidal Endothelial Cells

**Figure 4:**
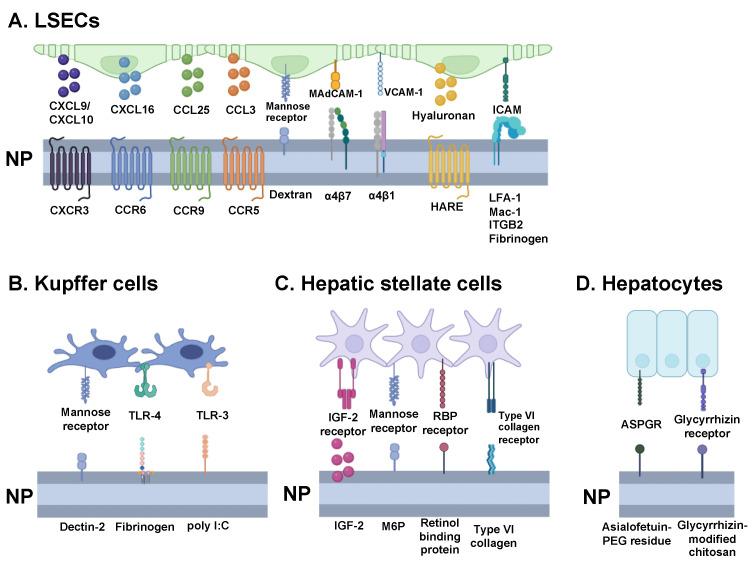
**Harnessing ligand-receptor interactions for nanoparticle design**. Overview of key ligands that can guide nanoparticles towards the different tolerogenic hepatic cells—(**A**) liver sinusoidal endothelial cells (LSECs), (**B**) Kupffer cells, (**C**) hepatic stellate cells, and (**D**) hepatocytes. By engineering nanoparticles with ligand receptors, they can more efficiently navigate toward the targeted cells to enhance tolerance induction efficacy. CXCL = C-X-C motif chemokine ligand; CXCR = C-X-C motif chemokine receptor; CCL = C-C motif chemokine ligand. CCR = C-C motif chemokine receptor; MAdCAM-1 = mucosal addressin cell adhesion molecule 1; VCAM = vascular cell adhesion protein; HARE = hyaluronan receptor for endocytosis; ICAM = intercellular adhesion molecule; LFA-1 = lymphocyte function-associated antigen 1; Mac-1 = macrophage-1 antigen; ITGB2 = integrin subunit beta 2; TLR = toll-like receptor; LPS = lipopolysaccharide; IGF = insulin-like growth factor; RBP = retinol-binding protein; ASPGR = asialoglycoprotein receptor; PEG = polyethylene glycol.

Liver sinusoidal endothelial cells form the lining of hepatic sinusoids, serving as the endothelial barrier between the sinusoidal blood and space of Dissé and allowing for the filtration of fluids and solutes [76]. To aid their role as the predominant scavenger cells of the liver, LSECs possess hyaluronan receptors, collagen alpha-chain receptors, scavenger receptors, mannose receptors, and Fcγ receptors, which equip them for endocytic clearance of varied macromolecules from circulation (Figure 4A) [77]. LSECs, thus, can quickly uptake foreign particles passing through the fenestrae, allowing them to exhibit a powerful clearance capacity of, for example, therapeutically delivered NPs [78].

In turn, this clearance capability enables LSECs to engage in a critical role in immunological tolerance. They serve as APCs and cross-present exogenous antigens to CD8^+^ T cells and induce antigen-specific CD4^+^FoxP3^+^ Tregs [79]. Notably, as demonstrated by Limmer et al., LSECs induce the proliferation of naive CD8^+^ T cells and, in their study, exhibited cross-presentation of OVA to CD8^+^ T cells in vivo [80]. Antigen presentation by LSECs to these T cells was found to induce immunological tolerance via apoptosis initiation, Treg induction, and the production of the anti-inflammatory cytokines IL-10 and TGF-β [78,80]. When compared to dendritic cells, LSECs have been observed to be over 100 times more efficient in antigen uptake in vitro and in vivo in mice and around twice as efficient in cross-presentation [81].

Regarding particle characteristics, LSECs have been observed to preferentially endocytose negatively charged and smaller particles [76,82]. For example, the polyanionic nature of polyaconitylated human serum albumin liposomes (Aco-HSAs) enabled preferential uptake by the liver (80% of administered dose) over other tissues and, specifically, LSECs over other hepatic cells when at a size of <100 nm [83]. This interaction was attributed to its interactions with the high abundance of scavenger cells on LSECs as removal of the negatively charged albumin peptide resulted in no LSEC uptake [83,84,85]. Interestingly, however, larger AcoHSAs in the range of 200–400 nm were increasingly endocytosed by Kupffer cells, supporting LSECs’ small size preference.

Adjacently, with NP-mediated gene delivery, Akhter et al. demonstrated the potential to deliver siRNA to LSECs via a multifunctional envelope-type nano device, modified with KLGR peptide as a surface ligand [85,86]. Similarly, hyaluronan-coated nanocapsules have also been successfully utilized to selectively target and deliver *Sleeping Beauty* transposon to LSECs, via the hyaluronan receptor for endocytosis (HARE) [87]. As will be expounded upon subsequently, Liu et al. demonstrated the potential for the NP induction of immunological tolerance in allergy pathologies using PLGA NPs to deliver OVA and Ara-h2 to LSECs [88,89].

### 3.2. Kupffer Cells

Adhered to LSECs, Kupffer cells are present within the lumen of liver sinusoids and function as the first line of immune cells in the liver. As the largest tissue macrophage population in the body, comprising approximately 80 to 90% of the population, KCs are involved in phagocytizing foreign particles and endotoxins inbound from the gastrointestinal tract via portal blood [90,91]. KCs possess a variety of pattern-recognition receptors (PRRs)—including mannose receptors, toll-like receptors (TLRs), and NOD-like receptors (NLRs)—that can recognize specific danger-associated molecular patterns (DAMPs) and pathogen-associated molecular patterns (PAMPs) and, thus, identify particles to remove from systemic circulation (Figure 4B) [92]. In turn, KCs engage in the phagocytosis of neutrophils, which, when subsequently cross-presented, have been observed to more frequently yield tolerogenic effects, rather than adaptive immune effects [93].

The precise mechanism of KCs to induce immunological tolerance is not yet fully understood; however, KCs have indeed been observed to suppress T-cell proliferation [94]. You et al. identified and examined three potential causes: T-cell apoptosis induction, regulatory T-cell (Treg) activation, or relatively insufficient stimulation by KCs [95]. Additional studies have supported the role of KCs in Fas/FasL-mediated T-cell apoptosis, via the upregulation of FasL by KCs [92,96].

One study demonstrated that NPs loaded with brain antigens were able to treat brain trauma after operations and induce peripheral tolerance by targeting KCs. In order to accomplish this, myelin-basic protein (MBP) was conjugated to polyvinyl alcohol (PVA)/beta poly β-amino ester (PBAE)/PLGA and ranged from 269.9 nm to 272.2 nm in size. The intermediate size of the NPs effectively achieved phagocytosis by KCs and prevented clearance by LSECs since the NPs were larger than the preferential size range for LSECs. Additionally, KCs’ mobility and favorable location within the space of Dissé allowed the NPs to be taken up by KCs [26].

Thus, these studies demonstrate the potential value of targeting KCs to induce immunological tolerance. Specifically, with respect to nanoparticle targeting, KCs’ various scavenger, toll-like, mannose, and Fc receptors present potential avenues for NP targeting and internalization [97]. As an example, mannosylated liposomes have been observed in mice to be effective in facilitating the delivery of muramyl dipeptide to KCs via mannose receptor-mediated endocytosis [98].

### 3.3. Hepatic Stellate Cells

Hepatic stellate cells, which are situated in the perisinusoidal space of Dissé in the liver, are known for their role in storing fat. These cells make up only 8% of liver cells; however, they store a significant amount of the body’s Vitamin A in lipid droplets (about 80%). Recent research by Winau et al. has shown that HSCs can also act as APCs. In their study, purified HSCs were pulsed with OVA-derived antigenic peptides and then cultured with CD4^+^ and CD8^+^ T cells. The HSCs were able to present the peptides to the T cells, indicating their ability to function as APCs [99,100]. Another study found that HSCs incubated with *Listeria monocytogenes* produced antigen-specific CD8^+^ T-cell proliferation, further supporting the notion that HSCs can function as APCs. Additionally, HSCs have been shown to successfully process and present exogenous antigens [101].

While specific nanoplatforms that target HSCs and promote immune tolerance have not been extensively researched, liposomal NPs have been used to target numerous cell surface receptors, including mannose-6-phosphate, insulin-like growth factor II, retinol-binding proteins, type VI collagen, and integrins (Figure 4C). These liposomal NPs have shown preferential uptake by HSCs and have been used to treat liver fibrosis in clinical trials [102]. For example, a vitamin-A-coupled liposomal NP containing siRNA targeting a retinol-binding protein was used in a phase 1b/2 clinical trial to treat liver fibrosis [103,104]. These NPs could potentially encapsulate food-specific antigens or autoantigens needed to treat allergies or autoimmune diseases.

Despite the potential therapeutic applications of HSCs, their main function remains controversial and is not well understood compared to other liver cells. Two reports suggest that HSCs have minimal APC functionality and only contribute to the tolerogenic environment of the liver by requiring the presence of dendritic cells, TGF-β, and retinoic acid to induce regulatory T-cell proliferation [105,106]. However, a possible mechanism to induce HSCs’ tolerogenic ability is to use NPs to deliver low levels of TGF-β along with dendritic cell chemoattractant [107]. It is important to note that since HSCs make up only a small fraction of liver cells and are located in an unfavorable position adjacent to endothelial cells in the space of Dissé, it is more likely that the majority of NPs will be internalized by LSECs and KCs [85].

### 3.4. Hepatocytes

Hepatocytes, often known for the metabolic role they play as the liver’s most populous cell, also help create a tolerogenic liver environment [108]. However, the exact mechanism of tolerance has not been made clear. Like HSCs, these large cells are situated past the space of Dissé and have microvilli that interact directly with circulating lymphocytes’ filopodia through endothelial fenestrations [109]. APC activity has been proposed, in which the hepatocyte MHC-1 activation of lymphocytes without co-stimulatory signals creates clonal deletion effects and ultimately tolerance against the cognate antigen [110]. For example, in transgenic mice with antigens expressed on both hepatocytes and within lymph nodes, when the primary activation of CD8^+^ T cells occurred in the liver due to the APC activity of hepatocytes instead of at lymph nodes, a tolerogenic phenotype of reduced half-life and impaired cytolytic function was observed [110]. The transient gene expression of MBP on hepatocytes also resulted in the generation of MBP-specific CD4^+^CD25^+^FoxP3^+^ Tregs to protect against EAE [111]. An alternative mechanism proposed that CD1-restricted interactions of natural killer T cells with hepatocytes generated IL-10-producing CD8^+^ T cells [112]. Therefore, while more research on the exact role that hepatocytes play in the liver’s tolerogenic capabilities is required, it is supported that hepatocyte targeting may provide antigen-specific preventive and therapeutic effects for autoimmunity and allergies.

Although there have been no identifiable reports of hepatocyte-targeting tolerance-inducing NPs, hepatocyte targeting with NPs has been met with variable success and numerous considerations. Nanoparticle size, for example, has demonstrated a significant impact on targeting since anatomical access to hepatocytes requires access to the space of Dissé via small fenestrae and avoidance of liver reticuloendothelial cells, which sequester most liver-reaching nanoparticles [24]. Therefore, a proposed <100 nm diameter has been suggested [24,113]. Hepatocyte-targeting has also been achieved with nanoparticle coating and compositions. Cholesterol-TWEEN 80 unilamellar vesicles and lipopeptide cKK-E12 showed preferential uptake by hepatocytes over sinusoidal cells while cKK-E12 lipoprotein NPs displayed gene silencing selectivity in hepatocytes over non-liver organs, liver endothelial cells, and liver leukocytes [113,114]. Hepatocyte-specific ligands have also been difficult to identify (Figure 4D). The most popularly implemented is the asialoglycoprotein receptor (ASGPR) due to its primary expression on hepatocytes, at ~1.8 million receptors/cell, and minimal expression on extrahepatic cells [14]. With a high affinity for N-acetylgalactosamine (GalNAc), galactose (Gal), and glucose, ASGPR has been tested for targeting with these and modified versions of these ligands, such as asialofetuin-PEG residue and galactosylated chitosan [115,116,117]. However, because Kupffer cells also demonstrate Gal/GalNAc scavenging, they could divert nanoparticles intended for hepatocytes [14]. Although less specific, glycyrrhizin/glycyrrhetinic acid receptors found on hepatocyte membranes, as well as kidney, stomach, and colon cells have also been targeted [118]. Glycyrrhizin-modified chitosan NPs, for example, have shown preferential endocytosis by hepatocytes over hepatic non-parenchymal cells [119]. Other commonly targeted receptors include transferrin, LDL, HDL, and folate receptors, amongst others [14,119]. However, many studies simply report liver accumulation rather than evidence of hepatocyte-specificity. Therefore, additional work is needed to characterize hepatocyte-specific uptake compared to other liver cells.

## 4. Disease-Specific Nanoplatforms for Liver-Targeting Tolerogenic Therapeutics

### 4.1. Anaphylaxis

The typical physiologic response to an allergen is a type I hypersensitivity reaction, resulting in the symptoms associated with an acute allergic reaction, such as hives, asthma, and dermatitis, among others. For some, it can even result in life-threatening anaphylaxis, characterized by difficulty breathing, nausea, vomiting, and loss of consciousness [11]. Anti-inflammatory options to treat acute allergic responses involve antihistamines, corticosteroids, and epinephrine. However, these immunosuppressive medications can inhibit immune surveillance and subsequently leave a person vulnerable to infections [64]. Another therapeutic option is allergen immunotherapy, which aims to reduce acute anaphylactic symptoms and prevent disease longevity. However, due to its recurrent administration via subcutaneous shots or sublingual tablets, it is highly involved and susceptible to patient noncompliance [89,120]. Thus, another immunotherapeutic approach is in development to address allergy symptoms—one that involves the use of nanoparticles and is advantageous due to its ease of production, scalability, and relative safety. 

To investigate whether liver-targeting NPs lend themselves to allergen tolerance, Liu et al. conducted an experiment encapsulating the OVA allergen in 230–290 nm PLGA NPs (NP^OVA^). Some NPs were decorated with APO B-100 peptide (ApoBP) ligand to target stabilin receptors on LSECs (NP^OVA^/ApoBPhi and NP^OVA^/ApoBPlo) (Table 1). These NPs were shown to accumulate in the liver and it was observed that prophylactic treatment with OVA NPs significantly decreased the IgE response, particularly for NP^OVA^/ApoBPhi, due to the higher concentration of surface ligands [88]. Additionally, pre-treatment of OVA-sensitized mice with both ApoBP-decorated OVA NPs reduced tissue inflammation to near-background levels and led to a significant increase in TGF-β, an anti-inflammatory cytokine. All OVA NP pre-treatments also resulted in reduced levels of IL-4, IL-5, and IL-13 and caused the generation of FoxP^3+^ Tregs in the lungs. Therefore, by minimizing allergic airway inflammation, PLGA NPs targeted to LSECs could provide a solution to inducing allergen-specific tolerance.

Capitalizing on LSECs’ role as APCs, NPs targeted to the liver can also encapsulate T-cell epitopes to generate Tregs. In a murine model, either the purified peanut allergen Ara-h2 or the Ara-h2 epitope was encapsulated and delivered via ~200 nm ApoBP-decorated PLGA NPs to determine which was more effective in reducing anaphylactic symptoms. The Ara-h2 epitope was successfully presented on the LSEC surface to induce differentiation into CD^4+^FoxP^3+^ Tregs. Conclusively, an oral challenge with crude peanut protein extract (CPPE) resulted in lower IgE levels and anaphylactic symptoms for both Ara-h2 pre-treatments but more so for the Ara-h2 epitope. Importantly, the prophylactic treatment was also shown to protect against anaphylaxis for at least 2 months [89]. The success of these liver-targeting, epitope-encapsulating nanoplatforms in minimizing the allergic response demonstrates that they can be an effective option in inducing tolerance for those with allergies.

**Figure 5:**
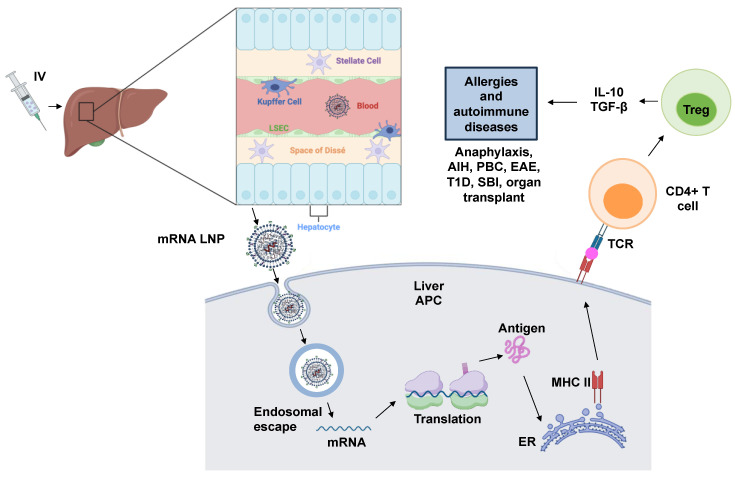
**Schematic of mRNA LNP platform**. Lipid nanoparticles (LNPs) are formulated by ionizable cationic lipids (e.g., MC3), helper lipids (e.g., DSPC and cholesterol), and PEG-lipids enveloping mRNA molecules and creating inverted micellar structures that protect mRNA from degradation and deliver mRNA to the cytosols for translation. After intravenous injection, the mRNA LNPs travel to the liver sinusoid through the hepatic artery and are taken up by residential antigen-presenting cells (APCs); then, the mRNA is released through endosomal escape and translated by ribosomes, synthesizing proteins as antigens. These antigens are presented to the cell surface after post-translational processing in the endoplasmic reticulum to generate epitopes that are loaded onto MHC-II molecules and presented on the APC surface. Naïve T cells recognize the epitopes binding to the MHC-II molecule and differentiate into regulatory T cells (Tregs) that express FoxP3 and checkpoint proteins (PD-1, PD-L1, CTLA-4, LAG-3, OX40, etc.) and secrete immunosuppressive cytokines, such as TGF-β and IL-10, etc. Tregs can induce immune tolerance and have the potential to treat allergies and autoimmune diseases, such as autoimmune hepatitis (AIH), primary biliary cholangitis (PBC), experimental autoimmune encephalomyelitis (EAE), type I diabetes (T1D), small bowel infarction (SBI), and solid organ transplant.

LNP usage in immune tolerance is a relatively new area of research; however, the initial results are encouraging. Studies have demonstrated the feasibility of mRNA LNPs in inducing antigen-specific tolerance in models of multiple sclerosis and peanut-induced anaphylaxis. Additionally, mRNA strands were designed to express T-cell epitopes derived from the peanut allergen protein Ara h2 and the LNPs were prepared with variations, including a mannose ligand on the particle surface to enhance LSEC targeting and uptake. The study demonstrated that the intravenous injection of the mRNA/LNP complex increased the generation of Tregs producing IL-10 in the spleen (Figure 5). Prophylactic administration of these particles also showed robust tolerogenic effects in an oral sensitization anaphylaxis model, reducing the physical manifestations of anaphylaxis and suppressing Th2-mediated immunity, allergen-specific IgE synthesis, and mast cell release. The study suggests that this mRNA epitope delivery approach to treat a peanut allergy holds promise and could be applied to other allergic disorders and autoimmune diseases [121].

### 4.2. Autoimmune Hepatitis

Autoimmune hepatitis (AIH) is a chronic T-cell-mediated disease that targets liver hepatocytes. As these liver cells are destroyed, the disease can cause inflammation, liver fibrosis, and eventual liver failure [122]. It is characterized by female preponderance, high serum transaminase levels, elevated IgG, and positive autoantibodies, as well as interface hepatitis [123]. The current standard of treatment involves the administration of a steroid, such as prednisolone, either as monotherapy or in combination with azathioprine, an immunosuppressive drug. While this has been the main treatment option for the past 50 years due to consistently favorable responses, some patients relapse after withdrawal and those who do not respond may progress to liver failure or cirrhosis [124].

As such, an alternative treatment involving the use of NPs to deliver steroids directly to the liver is indicated. By targeting the liver, a lower dosage can be administered and the occurrence of deleterious side effects can be reduced. In one study, dexamethasone, a steroid with 7.5 times the potency of prednisolone, was conjugated to Avidin-Nucleic-Acid-Nano-Assemblies (ANANAS) via an acid-labile biotin-hydrazone linker. This 132.9 ± 2.9 nm ANANAS-Hz-Dex conjugation was successfully biodistributed solely to KCs, potentially due to Dex’s hydrophobicity or ability to preferentially bind steroid receptors. This resulted in reduced liver inflammation markers. ANANAS themselves working as carriers was found to be neither toxic to cells nor pro-inflammatory and they remained in circulation for more than 2 h before being degraded [125].

Another therapeutic mechanism involves restoring peripheral tolerance towards host liver hepatocytes. While the autoantibodies associated with AIH are well-known, not all patients have them and their corresponding autoantigens are difficult to characterize. So far, the only unique autoantigens that have been discovered are SLA/LP/tRNP (Ser)Sec for AIH-1 and CYP2D6 and FTCD for AIH-2 [126]. In a study on AIH-2 mice, it was found that targeting FTCD with ex vivo expanded CXCR3+ Tregs was successful in restoring peripheral tolerance to FTCD autoantigens and causing remission in AIH mice [127].

Combining the nanoparticle delivery mechanism (ANANAS) with the method of targeting AIH autoantigens may provide a means to re-establish tolerance for those with AIH. As discussed by Richardson, et al., an efficient way to deliver autoantigens is through modified peptides that can engage CD4^+^ T cells but not pathogenic B cells or CD8^+^ T cells [126]. These peptides must be modeled after naturally occurring epitope conformations and bind directly to MHC-II in order to engage the relevant T cells [128]. When these peptides are presented via MHC-II to CD4^+^ T cells, they can differentiate into Tregs and restore peripheral tolerance.

### 4.3. Primary Biliary Cholangitis

Primary biliary cholangitis (PBC) is a chronic progressive autoimmune disorder caused by the destruction of the small intrahepatic bile ducts. This results in cholestasis, a buildup of bile in the liver that causes inflammation and eventual cirrhosis if left untreated. Much of the inflammation associated with PBC is thought to be CD8^+^ T-cell-mediated [129,130,131]. The first-line therapy is ursodeoxycholic acid (UDCA), which reduces the toxicity and concentration of bile acids in order to reduce liver inflammation [132]. However, 40% of PBC patients are unresponsive to UDCA and, despite the short-term alleviation of symptoms, current therapies are only able to delay, rather than cease disease progression [129].

Alternatively, NPs can not only prevent this progression but also diminish T-cell autoreactivity. In one study on autoimmune cholangitis (a PBC subtype), LSEC-targeting, 10 μM SIINFEKL peptide-conjugated NPs were administered intravenously to mice one day prior to OT-1-cell (SIINFEKL-specific CD8^+^ T-cell) transfer. It was found that the LSECs rapidly took up the NPs and cross-presented them on MHC-I to the CD8^+^ T cells, causing them to take on a more tolerogenic phenotype [1]. The pathogenic OT-1 cells in the SIINFEKL-treated mice were not able to infiltrate the liver and there was upregulation of PD-1 and downregulation of effector molecules, such as granzyme B, IFN-γ, and TNF. The ability of the SIINFEKL-NP to mitigate liver damage and reduce T-cell autoreactivity demonstrates the potential use of liver-targeting NPs to induce immune tolerance, even in CD8^+^ T-cell-mediated autoimmune diseases.

Beyond this, OT-1 cells homing to the spleen also displayed similar tolerogenic properties, indicating that CD8^+^ tolerance was induced not only in the liver but systemically. This is consistent with the finding that pMHC-II-based nanomedicines can not only blunt disease-specific autoantigens but also those of other liver autoimmune diseases (i.e., PDC NPs blunt PBC along with primary sclerosing cholangitis and AIH) [133]. Importantly, these nanomedicines accomplish this without impairing the immune system’s ability to generate antibodies, fight infections, or kill tumor cells.

To specifically induce tolerance in PBC, the autoantigen pyruvate dehydrogenase complex E2 subunit (PDC-E2), located on the biliary epithelium, is a good target. PDC-E2 is a well-defined autoantigen that can be delivered to the liver to be displayed on MHC-I [134]. Nanomedicines for PDC-E2 currently being developed include CNP-104 (Cour Pharmaceuticals, Skokie, IL, USA), which is in clinical trials, and ImmTOR (Selecta Biosciences, Watertown, MA, USA), which is in the process of seeking IND status as of November 2022 (Table 2) [135].

### 4.4. Multiple Sclerosis and EAE

Multiple Sclerosis (MS) is a chronic autoimmune disease in which self-reactive T cells attack the central nervous system axon myelin sheath or oligodendrocyte cell bodies, causing gliosis, plaque formation, and, eventually, neurological symptoms and functional disability [136,137]. Activated T cells have a variety of surface proteins that facilitate their extravasation across the blood–brain barrier and entrance into the central nervous system [138]. With no current cure for MS, treatments are aimed at slowing disease progression, preventing relapses, and managing symptoms. NPs are amongst several emerging immune-modulating approaches, which also include stem cells, DNA vaccines, and altered peptide ligands, aiming to destroy the self-reactive T cells and subsequently restore peripheral tolerance in MS patients [139].

Current NP therapies for MS are studied using experimental autoimmune encephalitis (EAE) animal models, which exhibit the key pathological features of MS, such as inflammation, demyelination, axonal loss, and gliosis [140]. EAE is artificially induced in animals via external immunization by injecting myelin proteins, such as myelin oligodendrocyte glycoprotein, MBP, and proteolipid protein [141].

Carambia et al. designed and tested the LSECs’ ability to induce immune tolerance in EAE murine models by using an iron oxide NP with a poly(maleic anhydride-alt-1-octadecene) coat (PMA1O) conjugated to a MBP or MOG antigen [82]. The PMA1O-NPs preferentially accumulated in LSECs due to their small size (20 nm) and negative charge. Iron oxide NPs conjugated to the MBP or MOG antigen were found to be effective in both prophylactically preventing and treating EAE with a single dose, demonstrating the PMA1O-NP’s successful therapeutic action and safety profile with no signs of adverse reaction or toxicity [82].

Casey et al. designed PLP-conjugated poly(lactide-co-glycolide) (PLG) NPs to successfully treat EAE, as shown by the increased expression of PD-L1 and the clonal deletion of T cells. The NPs were engineered to be in the 400–600 nm range and demonstrated preferential accumulation in the liver over other organs and, more specifically, in nearly all LSECs and KCs, 50% of HSCs, and 7% of hepatocytes. The strong association with both LSECs and KCs can be attributed to the emulsion-based manufacturing process that inherently creates a polydispersity of sizes, which is distributed to the different cells and their specific size preferences [142].

A study by Saito et al. explored different polymer compositions to influence APC phenotypes and Ag loading dose on NPs. The study examined the use of PLG-NP with poly(DL-lactide) (PLA) NP carriers and demonstrated superior reductions in the clinical scores of EAE and CD4^+^ T cells in the CNS when using the PLA-NP compared to PLG-NP. The NPs’ size ranged from 356 to 402 nm in order to target the liver, and they were readily phagocytized by KCs and LSECs. Moreover, the addition of a methyl group in the lactide made the PLA more hydrophobic than PLG, which allowed for a stronger association with liver APCs. Analysis with flow cytometry showed a greater phagocytosis of PLA-NPs in the KCs, LSECs, and liver dendritic cells than PLG-NPs and led to a decreased expression of CD86 in KC and LSECs as a result of anergy and the inactivation of CD4^+^ T cells. Thus, the slower degrading and higher antigen load of PLA-NP is an effective NP design to treat EAE in mice [143].

A recent study by Krienke et al. demonstrated the viability of using mRNA lipid nanoparticle (LNP) vaccines for autoimmune diseases. They showed that vaccination with modified mRNA (m1Ψ mRNA-LPX) encapsulated in LNPs induced antigen-specific tolerance in the EAE model. This was achieved by facilitating antigen presentation by CD11c^+^ APCs in a non-inflammatory environment. The findings highlight the potential of mRNA-based vaccines to induce immune tolerance in autoimmune diseases like MS [144].

The use of tolerogenic nanoparticles to induce immune tolerance via liver APCs for the treatment and prevention of EAE in murine models has been promising, especially with improvements in NP designs enabling improved targeting of the liver APCs and treatment with lower doses. Although EAE remains the standard animal model for MS, further studies are warranted as treatment and therapies designed in animal models may not reflect the same complexity in humans.

### 4.5. Type I Diabetes

Type I diabetes (T1D) is caused by the autoimmune destruction of insulin-producing pancreatic ß islet cells. This is driven by CD4^+^ and CD8^+^ T cells, macrophages, and B-cell infiltration and results in insulin deficiency and hyperglycemia [145,146]. Without careful monitoring of blood glucose levels, T1D leads to major complications including blindness, renal failure, stroke, and even death. The current standard of care for T1D is strict dietary control, exogenous insulin replacement therapy, and constant blood glucose monitoring. While beneficial, these modalities require diligent patient involvement and fail to provide long-term tolerance that allows for ß islet cell function.

In novel therapeutic approaches to T1D, it would be more effective to address the cause of ß cell destruction. In a study on NOD.SCID mice by Prasad et al., the p31 mimotope peptide was coupled to negatively charged PLG NPs (p31-PLG) and injected intravenously, followed by the transfer of diabetogenic CD4^+^ BDC2.5 transgenic T cells that could be activated by p31. Compared to control mice, the onset of T1D was delayed in p31-PLG-treated mice [147]. Tolerance was shown to be peptide-specific, as only p31-PLG transfusion induced tolerance in the MHC-II-restricted BDC2.5 T cells while a different MHC-I-restricted mimotope peptide failed to do so. Peripheral tolerance from p31-PLG was initiated via several pathways, involving negative co-stimulatory molecules, such as CTLA-4 and PD-1, as well as inducing peptide-specific Tregs. This resulted in the retention of diabetogenic BDC2.5 Tg cells in the spleen instead of migration to the pancreas, effectively limiting infiltration and pro-inflammatory cytokine production within ß islet cells. Accordingly, the administration of p31-PLG was shown to block and reverse ß cell destruction and restore tolerance. In another adoptive transfer experiment by Jamison et al., a 2.5HIP-PLG NP infusion targeted BDC2.5 T cells, inducing anergy in effector T cells and, subsequently, reducing pro-inflammatory cytokine production. The increase in the ratio of FoxP3^+^ Tregs to IFN-γ^+^ T cells demonstrated an effective reprogramming of diabetogenic T cells via the use of NPs [148].

**Table 1:** Liver-targeting tolerogenic NP design, size, and antigen for autoimmunity and allergies. All modalities were administered via an intravenous route.

Disease	Nanocarrier	Size (nm)	Antigen
Egg allergy	PLGA NP ApoBP-conjugated PLGA NP Mannose-coated PLGA NP [66]	200–300	OVA
General allergy	PLGA NP ApoBP-coated PLGA NP [66]	∼230–250	OVA + curcumin OVA + rapamycin OVA323–339OVA257–264 epitopes
Peanut allergy	ApoBP-coated PLGA NP [66]	200–300	Ara-h2 epitope
AIH (Autoimmune hepatitis)	ANANAS NP with biotin-hydrazone linker [125]	132.9 ± 2.9	Dexamethasone (steroid)
PBC	PLGA NP [1]	10,000	SIINFEKL peptide
EAE	PLGA and PLA NPs [143]	356–402	PLP 139–151
	PLP NP [142]	400–600	PLP 139–151
	PMAcOD-coated iron oxide NP [82]	20	MBP MOG
T1D	PLG/PEMA [147]	500	p31 NRPA7 MOG35–55 p31-NRPA7-InsB9–23
	PLG [148]	500	2.5 hybrid insulin peptide
	IDLV + ICLV [149]	N/A	Immunodominant epitope of insulin [insulin B chain 9–23 (InsB9–23)] transgene
SBI	PVA/PBAE/PLGA [26]	270–272	MBP Brain protein

**Table 2:** Pre- and clinical trials for liver-targeting tolerogenic nanotherapeutic platforms.

Disease	Nanotechnology	Type	Phase	Assessment	Status
Peanut allergy	CNP-201 (Cour Pharmaceuticals) [135]	Purified peanut extract (PPE) in PLGA NP	Two-part Phase 1b/2a, First-in-Human (FIH) Trial	Safety, tolerability, pharmacodynamics, and efficacy of multiple ascending doses of CNP-201	Study start date: 14 March 2022 Est study completion date: 1 December 2024
PBC	CNP-104 (Cour Pharmaceuticals) [135]	PDC-E2 peptide in PLGA NP	Phase 2a FIH Trial	Safety, tolerability, pharmacodynamics, and efficacy of CNP-104 in subjects who are unresponsive to UDCA and/or OCA	Study start date: 25 January 2022 Est study completion date: 30 December 2025
PBC	ImmTOR (Selecta Biosciences) [135]	Co-administration of ImmTOR with PDC-E2	N/A	N/A	As of 3 November 2022, continuing IND-enabling work

In order to test the tolerogenic ability of the liver, Akbarpour et al. treated T1D in mice by injecting an integrase-competent lentiviral vector (ICLV) that targeted the expression of the transgene immunodominant epitope of insulin (InsB9–23) in hepatocytes. The ICLV treatment, along with a single dose of the anti-CD3 monoclonal antibody, protected 90% of mice from developing T1D and induced a state of tolerance via an upregulation of FoxP3^+^ Tregs [149]. InsB9–23-specific Tregs were shown to first be induced in the liver before spreading to the pancreatic lymph nodes where the antigen was presented to T cells. The study demonstrated the ability to induce antigen-specific tolerance in T1D via gene transfer to hepatocytes.

There are several challenges to developing treatments for immune tolerance to T1D in humans, one being autoantigen target selection. Several autoantigens are associated with T1D in humans, including the C peptide of proinsulin, ß cell antigen insulin, islet-glucose-6-phosphatase catalytic subunit-related protein, glutamic acid decarboxylase 65, insulinoma-associated protein 2, and zinc transporter 8. However, no single epitope is pathogenic and multiple autoantigens are likely targeted in T1D, making it difficult to translate mice NP models to humans [145]. Additionally, timing is an important concern in type 1 diabetics as some may have limited residual islet cell mass by the time they are diagnosed, hampering the potential use of tolerance induction. In those cases, T1D-Ag-PLG particles would need to be used alongside some form of islet cell replacement, such as transplantation [150,151]. As population screening efforts for T1D risk improve and earlier detection increases, PLG NPs provide a strategy to induce tolerance and either prevent the onset of T1D or limit its effects [152].

### 4.6. Surgical Brain Injury

Surgical brain injury (SBI) is an autoimmune reaction in which the blood–brain barrier is compromised after a neurosurgical operation, causing exposure of brain antigens to the body’s systemic immune system [153]. Normally, the highly selective blood–brain barrier creates an environment where brain cells do not encounter blood-borne pathogens and immune cells. As a result, the body mounts immune responses to these perceived “foreign” brain antigens and sequesters inflammatory molecules like cytokines and chemokines. This aggravates neuronal damage, brain edema, mitochondrial dysfunction, and oxidative stress and can cause other neurological deficits [26,154]. Current treatments for SBI include anti-inflammatory steroid hormones, dehydration to reduce intracranial pressure, non-specific diuretics, and immunosuppressive agents [155,156].

Tian et al. investigated treating SBI by using liver bio-targeting NPs loaded with brain antigens to develop immune tolerance. They engineered PVA/PBAE/PLGA NPs loaded with brain protein and MBP to match the size range that would allow phagocytosis by KCs, as discussed earlier. These NPs were injected intravenously into murine models, confirmed to accumulate in the liver, and shown to decrease the levels of helper T cells, indicating the downregulation of immune responses to brain antigens. Moreover, concentrations of pro-inflammatory cytokine IL-2 decreased, anti-inflammatory cytokine IL-4 increased, and immune-suppressing cytokine TGF-β1 increased [56]. Thus, this study offered a new method of treatment for SBI that allows for a high degree of liver targeting and effectively produces immune tolerance.

### 4.7. Solid Organ Transplant

For end-stage liver disease patients, solid organ transplant is the standard of care. However, this often results in acute or chronic autoimmune rejection of the transplanted organ and potential complications include heart failure, stroke, and infection [157]. In 2021, 9234 liver transplants were performed in the U.S., with 19–24% of transplant recipients experiencing at least one rejection episode within a year [158]. Patients who receive a solid organ transplant must remain on maintenance immunosuppression for the rest of their lives to prevent graft loss and there is often a fine line to avoid the risks of underimmunosuppression (rejection) and overimmunosuppression (infection) [159]. Other adverse events associated with chronic immunosuppressive use include nephrotoxicity, wound healing impairment, skin malignancy, thrombocytopenia, and lymphomas [160].

An alternative approach involves pre-conditioning the graft prior to transplantation in order to minimize potential immune activation. The advantage of this ex vivo perfusion mechanism is that accurate targeting can be achieved quickly with fewer immunological or physiologic factors to consider [160]. A proposed target is the modification of donor graft vasculature to reduce the expression of non-self MHCs. In a study by Cui, et al., non-self MHC II siRNA were loaded into poly(amine-co-ester) NPs via ex vivo perfusion, resulting in decreased MHC II expression, T-cell infiltration, and T-cell-mediated inflammation after transplantation [161]. However, these effects only last a span of weeks; thus, it is mainly useful for preventing acute transplant rejection. For the prevention of chronic transplant rejection, the immunosuppressant rapamycin, which is typically used in solid organ transplants, could be used to pre-treat grafts. Zhu et al. found that using targeted rapamycin micelles in ex vivo treated grafts required a 10-fold lower dose, compared to free rapamycin, to achieve the same suppressive effects [162]. The unique ability to pre-condition grafts using nanoparticle delivery methods has the potential to minimize peri-transplant adverse effects and improve post-transplant outcomes.

## 5. Challenges in Human Translation and Perspectives

Current therapeutic interventions for allergies and autoimmune diseases, such as general immunosuppressants and immunomodulators, lack specificity and suppress protective immune responses in addition to the intended autoreactive ones. Other antigen-specific therapies not utilizing nanoparticles have faced challenges due to their lack of targeting specificity within the body, resulting in broad side effects and higher-than-necessary therapeutic dosages [2,163]. As a result, a targeted approach that utilizes the body’s intrinsic tolerogenic mechanisms to reverse antigen-specific disease pathologies without compromising the immune system would be a major advancement in the field [2]. Fortunately, nanoparticles possess highly versatile physicochemical properties that make them an attractive solution for selectively and efficiently inducing immune tolerance in the liver for the treatment of allergies and autoimmune diseases.

The immunosuppressive properties of NPs depend on their size, shape, zeta potential, composition, ligands, and other modifications, allowing for adept engineering to provide various therapeutic advantages, such as passive targeting, active targeting, and drug delivery. The use of cutting-edge technology has enabled the successful engineering of natural and synthetic polymers to deliver drugs with improved efficiency, influencing pharmacokinetics, distribution in vivo, clearance pathways, and interactions with liver cells. NPs can be administered through various routes, including intravenous, intraperitoneal, oral, and inhalation. Among these, intravenous administration is the most common as it provides an instantaneous response and allows for NP accumulation in the liver, decreasing the required dosage for effective treatment. However, intravenous administration has some drawbacks, such as the need for healthcare professional assistance, high invasiveness, high cost, and decreased accessibility [164].

Combination therapy with tolerogenic NPs, in which liver-targeting NPs are used in conjunction with NPs delivering pharmaceutical agents to realize synergistic tolerance, is one way to take further advantage of NPs’ versatility. For example, NPs encapsulating MOG and IL-10 achieved prophylactic and therapeutic intervention in a chronic progressive EAE model [165]. Another study described the use of hybrid particles to encapsulate TGF-β surface protein constructs, such as MHC-I and -II multimers, presenting MBP peptides to autoreactive T cells, anti-Fas mAb and a recombinant PD-L1-Fc construct for apoptosis induction in autoreactive T cells, or CD47-Fc for inhibiting NP sequestration and prolonging in vivo half-life [166]. With this information, the construction of hybrid platforms that combine LSEC targeting as well as pharmaceutical enhancers of APC tolerogenic activity, enhancement of Treg generation, and stable FoxP3^+^ expression should be considered for treating a range of antigen-specific immune disorders characterized by overactive immune function [66].

The expansion of liver-targeting tolerogenic NPs to the diversity of NP composition materials is another avenue for innovation. As described, most current research on these existing NPs utilizes PLGA as the material of choice for its biostability and release rate efficiency control that allow for non-toxic liver accumulation [26]. Although metal- and carbon-based nanomaterials have shown successful immunotherapeutic effects as previously described, their bioaccumulation and biotoxicity must be addressed. LNPs are promising, in particular, for their diverse loading capabilities (siRNA, mRNA, DNA, peptides, etc.), ease of surface modification, and propensity toward liver accumulation [57]. Additionally, the synthesis of new lipids/formulations and the use of new manufacturing techniques have advanced the field of LNPs to improve liver targeting, e.g., reinforced particle stability, enhanced cargo loading, and reduced unintended opsonization, as evidenced by the ONPATTRO^®^ design [167]. The incorporation of immune tolerogenic mechanisms, such as antigen epitopes and ligands targeting specific hepatic cells, into these advanced LNP designs, may, therefore, expand therapeutic approaches to autoimmunity [121].

Conversely, the integration of different ligands into the design of tolerogenic NPs can enhance liver targeting. Ligands targeting receptors concentrated on specific liver cells, such as hyaluronan receptors, mannose receptors, retinol-binding proteins, and ASGPR, can be conjugated onto the surface of tolerogenic NPs to prevent bioaccumulation and action in non-tolerogenic organs or reduce the given dosage. A biomimetic design can achieve similar results, as illustrated by recombinant HDL NPs, which were preferentially taken up by the liver [168]. To magnify these approaches, however, additional biochemical research may be required to identify additional target ligands and the profile of features (e.g., size, charge, shape, and composition) that the intended liver cells are predisposed to. This research is especially important when targeting NPs to cells beyond LSECs, whose role as the major scavenger cells makes them the physiological default for the clearance of foreign substances that reach the liver [78]. By overcoming this LSEC “hurdle”, selective nanomedicine drug delivery can also be used to activate multiple of these hepatic APCs and create broad, synergistic effects of antigen-specific tolerance via multiple mechanisms [169].

Similarly, the conjugation of activating ligands (e.g., Ah-2, Fas monoclonal Ab, CD22 glycan) and blocking ligands (e.g., macrophage membrane antigens, MHC-I, MHC-II), which have been shown to enhance a tolerogenic state and suppress inflammatory signals, onto tolerogenic NPs can further augment the antigen epitope for reinforced antigen-specific tolerance induction.

With these improvements in targeting and therapeutic mechanistic action, liver-targeting tolerogenic NPs can be implemented in the treatment of other allergies and autoimmune disorders as well. Some examples of antigen epitopes or peptides to prevent allergies include B-lactoglobulin (whey protein allergy), mugwort pollen (pollen allergy), Fel d 1 (cat allergy), Der s 1 (house dust mite allergy), and cashew nut extract (cashew allergy) [170,171,172,173,174,175]. Examples of future antigen epitopes or peptides for the treatment of autoimmunity include gliadin (celiac disease), type II collagen (collagen-induced arthritis), alpha-glucosidase (Pompe disease), and Factor VIII (hemophilia A) [74,176,177,178]. By targeting these studied NPs and their cargo to the liver, improvements in their therapeutic efficiency can overcome some of their current limitations. One such obstacle to the widespread clinical application of this approach, however, involves identifying and verifying relevant MHC alleles, antigens, and epitopes for a diverse human population.

Although the unique properties of NPs provide a range of clinically desired features, such as higher drug loading and increased bioavailability, there are still many improvements to the vehicle that need to be made. The primary challenge in applying NPs to humans lies in their potential for toxicological effects on the human body. The immunosuppressive and targeting capabilities of NPs are intricately connected to their size, shape, zeta potential, composition, crystalline structure, and dose, which similarly influence the toxicity of NPs. For example, the small surface area to volume ratio, which is, on one hand, biomedically beneficial, can also give rise to unexpected toxicities [179]. Notably, in vitro studies of PLGA and TiO_2_ NPs demonstrated that smaller particles and higher doses increased reactive oxygenated species generation and TNF-α release in cells [180].

The interaction between NPs and biological cells is very important; understanding the intricate pathways and mechanisms by which NPs interact with cells is critical for optimizing the design of delivery systems. Over the past two decades, the field of nanotoxicology has seen significant advancements, particularly in understanding the complex interactions at the nano/bio interface. The initial encounter between NPs and biological systems, e.g., proteins, lipids, nucleic acids, etc., is a dynamic interplay of physicochemical properties that triggers the formation of a protein corona, masking and modifying the NP’s original characteristics [181]. The composition and evolution of this corona significantly influence the subsequent interaction with the cell membrane and determine the NP’s fate [182]. Therefore when delving deeper into the NP cellular responses, current insights show that major mechanisms of NP-induced toxicity include reactive oxygen species accumulation and oxidative stress, the dissolution and release of toxic metal ions, cationic injury to the cell membrane and organelles, membrane lytic and pro-fibrogenic responses to surface reactivity, inflammasome activation and inflammation, the photoactivation and influence of bandgap, DNA damage, cell cycle disruption, and epigenetic regulation [183,184,185]. Importantly, NPs have displayed the capability to traverse the blood–brain barrier and blood–testis/blood–follicle barrier, accumulating in the brain, ovaries, and testes, causing neuronal and reproductive organ dysfunction [183,186]. Thus, despite the promising potential for NPs in innovative medical treatments, it is crucial not to disregard their potential side effects. Currently, most published studies showcasing the encapsulation of clinical drugs with NPs are confined to the pre-clinical stage; only a limited number successfully progress through clinical trials to attain commercial viability [183]. This trend underscores the imperative for further research to comprehensively address issues related to NP toxicity and formulations, particularly in the context of large mammalian and non-human primate models, to ultimately mitigate potential adverse effects [179].

Another large barrier to current commercial success is the expenses. The complexity in NP synthesis, including condensation, evaporation, combustion, and hydro-thermal-based synthesis, creates a high cost of manufacturing while some of the chemical synthesis methods, including thermal decomposition and etching, pose environmental risks that only further increase costs [187].

Given the broad advancements in nanomedicine technology, liver-targeting NPs with antigen-specific tolerogenic properties have many avenues for improvement and expansion. With further research, these targeted NPs have the potential to provide a targeted approach that exploits the body’s intrinsic tolerogenic mechanisms to reverse antigen-specific disease pathologies without compromising the immune system, representing a major advancement in therapeutic interventions for allergies and autoimmunity.

## 6. Conclusions and Outlook

In conclusion, liver-targeting NPs have emerged as a promising platform for inducing immune tolerance, leveraging the distinctive tolerogenic properties of the liver and the adaptable physicochemical characteristics of nanoparticles. This comprehensive review evaluates the crucial design parameters essential for achieving cell-specific targeting and for the induction of tolerogenic responses by nanoplatforms tailored to address the four key immunogenic hepatic cells. The examination of various therapeutic strategies utilizing nanoparticles for the treatment of diverse allergies and autoimmune disorders underscores the broad applicability of this approach in immunology. 

Despite the immense potential, there are challenges associated with employing NPs in this context, including issues related to toxicity, formulation, manufacturing costs, etc. However, further research and innovation can overcome such current limitations to ensure the safe and effective utilization of nanoparticles for immune tolerance induction. Future directions include integrating pharmaceuticals and expanding on NP composition materials beyond the commonly used polymer- and/or lipid-based materials into other combinatorial formulations, such as the emerging individually designed mRNA-encapsulated lipid nanoparticles [188]. The mRNA LNP platform is notably flexible, rapid in development, and cost-efficient [15]. It can facilitate the customization of antigen or epitope designs tailored to individual patients [121,188]. Moreover, it provides the opportunities to amalgamate diverse antigens or epitopes within a single formulation. Ultimately, the evolving landscape of liver-targeting NPs holds promise for advancing therapeutic interventions in immunologic diseases and offering more targeted and efficacious treatments for allergies and autoimmune disorders.
